# Early response to upfront neoadjuvant chemotherapy (CAPOX) alone in low- and intermediate-risk rectal cancer: a single-arm phase II trial

**DOI:** 10.1093/bjs/znab388

**Published:** 2021-11-18

**Authors:** Xiangbing Deng, Qingbin Wu, Liang Bi, Yongyang Yu, Shuo Huang, Du He, Bing Wu, Hongfeng Gou, Wenjian Meng, Meng Qiu, Yazhou He, Ziqiang Wang

**Affiliations:** Department of Gastrointestinal Surgery, West China Hospital, Sichuan University, Chengdu, China; Department of Gastrointestinal Surgery, West China Hospital, Sichuan University, Chengdu, China; Department of Gastrointestinal Surgery, West China Hospital, Sichuan University, Chengdu, China; Department of Colorectal Surgery, Gansu Provincial Hospital, Lanzhou, China; Department of Gastrointestinal Surgery, West China Hospital, Sichuan University, Chengdu, China; Department of Gastrointestinal Surgery, West China Hospital, Sichuan University, Chengdu, China; Department of Pathology, West China Hospital, Sichuan University, Chengdu, China; Department of Radiology, West China Hospital, Sichuan University, Chengdu, China; Department of Abdominal Cancer, West China Hospital, Sichuan University, Chengdu, China; Department of Gastrointestinal Surgery, West China Hospital, Sichuan University, Chengdu, China; Department of Abdominal Cancer, West China Hospital, Sichuan University, Chengdu, China; Department of Oncology, West China School of Public Health and West China Fourth Hospital, Sichuan University, Chengdu, China; Department of Gastrointestinal Surgery, West China Hospital, Sichuan University, Chengdu, China

## Abstract

**Background:**

With local recurrence of rectal cancer continuing to decrease, distant recurrence is becoming a major concern, especially for patients with low- and intermediate-risk stage II/III rectal cancer. Therefore, a new treatment strategy is warranted for these patients. This single-arm phase II trial aimed to assess the effect of neoadjuvant chemotherapy (NCT) in low- and intermediate-risk stage II/III rectal cancer and explore candidate radiological and clinical parameters for early prediction of tumour response after two cycles of CAPOX.

**Methods:**

Patients with mid–low stage II/III rectal cancer with low and intermediate risk were examined. The primary outcome was defined as a clinicopathological response by integrating tumour longitudinal length reduction (TLLR) on MRI into pathological tumour regression grade (TRG). After completing NCT, patients with TRG0–2 and TRG3 with a TLLR rate greater than 30 per cent were considered to be responders. Secondary outcomes included pathological complete response (pCR), adverse events and local and distant recurrence.

**Results:**

This study enrolled 61 eligible patients. No patient was converted to neoadjuvant chemoradiotherapy owing to tumour progression. The clinicopathological response and pCR rates were 78.7 and 21.3 per cent respectively. After two cycles of CAPOX, TLLR, TRG on MRI, and mucosal lesion regression grade on endoscopy had potential discriminative ability (area under the curve greater than 0.7) for predicting both clinicopathological and pathological response.

**Conclusion:**

NCT alone achieves good tumour response rates in patients with low- and intermediate-risk stage II/III rectal cancer, and predicting tumour response to NCT is feasible at an early treatment phase.

**Registration number:**

NCT03666442 (http://www.clinicaltrials.gov).

## Teaser

Neoadjuvant chemotherapy alone achieves desirable tumour response rates in low- and intermediate-risk stage II/III rectal cancer. Tumour longitudinal length reduction, tumour regression grade on MRI, and mucosa lesion regression grade on endoscopy are promising for early prediction of both clinicopathological and pathological response.

## Introduction

The introduction of neoadjuvant chemoradiotherapy (NCRT) has significantly improved disease management in patients with locally advanced rectal cancer; however, it is yet to improve the overall survival rate^1–3^. Previous studies observed low local recurrence rates (LRRs) of less than 5 per cent in patients without high-risk factors who underwent total mesorectal excision (TME) surgery alone^4^^,^^5^. Moreover, the authors’ previous randomized trial showed that patients with low-risk stage II/III rectal cancer had an even lower 3-year LRR of 3 per cent, and no significant benefit to LRR was found with the addition of radiotherapy^6^. In fact, the European Society of Medical Oncology (ESMO) recommends stratifying treatment of rectal cancer patients and avoiding neoadjuvant radiotherapy in intermediate- or low-risk patients, provided that high-quality surgery is assured^7^.

With local recurrence posing less of a threat, distant recurrence is the main concern^8^. There are a number of factors that can lead to an elevated risk of distant recurrence, such as a delay in postoperative chemotherapy, poor compliance with systemic treatment after surgery, or lack of evaluative approaches to treatment response^9^^,^^10^. These are all compelling reasons for the application of neoadjuvant chemotherapy (NCT) to reduce distant recurrence. Exploratory studies assessing the feasibility of NCT in a mix of patients with low- and high-risk rectal cancer reported varied downstaging rates between 48.8 and 80 per cent and pathological complete response (pCR) rates of 4 to 13 per cent^11–13^. A recent trial observed unacceptably high LLR of 11.5 per cent for good responders and 34 per cent for poor responders in patients with high-risk stage II/III rectal cancer^14^, indicating that NCT alone might be applicable only for low- and intermediate-risk patients.

As NCT could be less effective in downstaging primary tumours, it is imperative to identify those with poor response in the early phase of NCT with timely change to alternative treatment. Ongoing efforts (trials number NCT01515787 and NCT02288195) aim at evaluating tumour response after completing NCT. Only one study with 15 enrolled patients conducted a response assessment within 6 weeks of NCT based solely on PET-CT characteristics^15^. There has been a dearth of investigations using comprehensive clinical and radiographic features for early response assessment. Thus, this single-arm phase II trial aimed to estimate the effect of NCT in low- and intermediate-risk stage II/III rectal cancer and explore candidate radiological and clinical parameters for early prediction of tumour response (after 6 weeks) to NCT.

## Methods

### Study setting and patient recruitment

This was a prospective, open-label, single-arm phase II trial enrolling patients with mid–low stage II/III rectal cancer with low and intermediate risk. The trial was approved by the Ethics Committee of the West China Hospital and registered at clinicaltrials.gov (registration number NCT03666442). Informed consent was signed by each participant before enrolment.

Low and intermediate risk were defined according to the ESMO guideline^7^: cT3a-bN0-1M0 for low rectal cancer and cT3a-c/T4aN0-1M0 for middle rectal cancer, without threatened mesorectal fascia. A T3 stage was assigned only if both MRI and transanal ultrasound (TRUS) agreed with each other to avoid over-staging. N2 was defined as the presence of more than three lymph nodes with a short axis of 8 mm or greater. Patients who were scheduled for extralevator abdominal peritoneal excision (ELAPE) because of anal sphincter invasion were deemed eligible, as an extramural invasion of less than 5 mm with a negative margin was considered achievable with ELAPE. Detailed inclusion and exclusion criteria are described in *Table S1*. All eligible patients underwent colonoscopy, chest–abdominopelvic CT, rectal high-definition MRI, TRUS and digital examination before treatment.

### Intervention

#### Neoadjuvant treatments

Four cycles of CAPOX (oxaliplatin 130 mg/m^2^, once daily on day 1, every 21 days; capecitabine 1000 mg/m^2^, twice daily on days 1 to 14, every 21 days) were administered. After two cycles, patients were advised to receive standard NCRT (50.4 Gy/28 f/1.8 Gy radiation therapy for 5 days per week with capecitabine 825 mg/m^2^ twice daily concurrently) if the tumour was determined as progressive (tumour longitudinal size increase greater than 20 per cent). Adverse events of chemotherapy were documented according to the National Cancer Institute Common Toxicity Criteria^16^, version 4.03.

#### Surgical procedure

TME surgery was performed by experienced colorectal surgeons 2–4 weeks after completion of NCT or 6–8 weeks after completion of NCRT. Preventive ileostomy was not commonly performed. Selective lateral pelvic lymphadenectomy was performed if the short axis of the lateral lymph node was greater than 5 mm.

### Outcome measures

Initial (before NCT), early (after two cycles) and preoperative evaluations were conducted based on findings from colonoscopy, chest–abdominopelvic CT, rectal high-definition MRI and TRUS (*Fig. S1a*). Patients converted to NCRT were assessed 5–6 weeks after radiation. In the initial and early evaluation, characteristics including tumour longitudinal length (TLL) on MRI, tumour regression grade (TRG) on MRI (mrTRG), mucosal lesion regression grade (MLRG) on endoscopy, tumour thickness on TRUS, and MRI textures, were reported. The TLL was defined as the distance between the upper and lower bounds of the tumour parallel to the axis of the intestine (illustrated in *Fig. S1b*). The tumour longitudinal length reduction (TLLR) rate measured the shrunken percentage of TLL at early evaluation compared with that at the initial evaluation. The mrTRG was assessed based on T2W1 images according to Battersby’s study^17^. During endoscopy, a sterile non-woven cloth (1 cm in diameter) was placed beside the tumour to assess the MLRG (*Fig. S1c*). Tumour thickness was measured by the same ultrasound diagnostic using TRUS at different evaluations. MRI textures were extracted using Pyradiomics (vision 2.2.0 https://pyradiomics.readthedocs.io) on unenhanced axial T2W images by manually delineating the region of interest that ran through all consecutive slices. One hundred texture features were recorded, including first-order statistics (18 features), shape descriptors (14 features), grey-level co-occurrence matrix (36 features), grey-level run length matrix (16 features), and grey-level size zone matrix (16 features)^18^. Surgical specimens were evaluated by experienced pathologists following the American Joint Committee on Cancer and College of American Pathologists tumour regression grade (AJCC/CAP TRG) system^19^.

A clinicopathological response was defined as the primary endpoint by integrating TLLR on MRI as recommended by the Response Evaluation Criteria in Solid Tumours (RESIST, version 1.1)^20^ and previous investigations^21^. A clinical partial response (PR) was defined as a greater than 30 per cent reduction in TLL. An increase of greater than 20 per cent in TLL was considered to be clinical progressive disease (PD). Otherwise, the tumour was diagnosed as clinical stable disease. Clinicopathological responses were assigned to patients with pathological TRG (pTRG) 0–2 or pTRG3 with clinical PR. The secondary endpoints included the pathological complete response (pCR) rate, adverse events, and local and distant recurrence.

### Statistical analysis

As a single-arm, phase II trial, the sample size was estimated based on the hypothesis of a non-inferior response rate of NCT compared with NCRT. The clinicopathological response rate of NCRT was assumed to be 80 per cent, and a non-inferiority margin of 10 per cent was adopted. Fifty-eight patients were required with α  = 0.05, and β =  0.80. Finally, it was planned than 60 patients would be recruited.

The patients’ baseline characteristics were summarized using descriptive statistics. The distribution of categorical parameters was compared using a two-sided Fisher’s exact test. Receiver operating characteristic curves were created and the area under the curve (AUC) was calculated along with 95 per cent confidence intervals to evaluate the predictive performance of each variable. Parameters for multivariable modelling were selected using a least absolute shrinkage and selection operator (LASSO), including the 100 MRI texture features along with clinicopathological characteristics. Cross-validation was used to adjust for potential overoptimism of the estimated AUC. Statistical significance was set at *P *<* *0.050. Statistical analysis was performed using R software (version 3.4.1. R Foundation for Statistical Computing, Vienna, Austria.).

## Results

### Patient characteristics

**Fig. 1:**
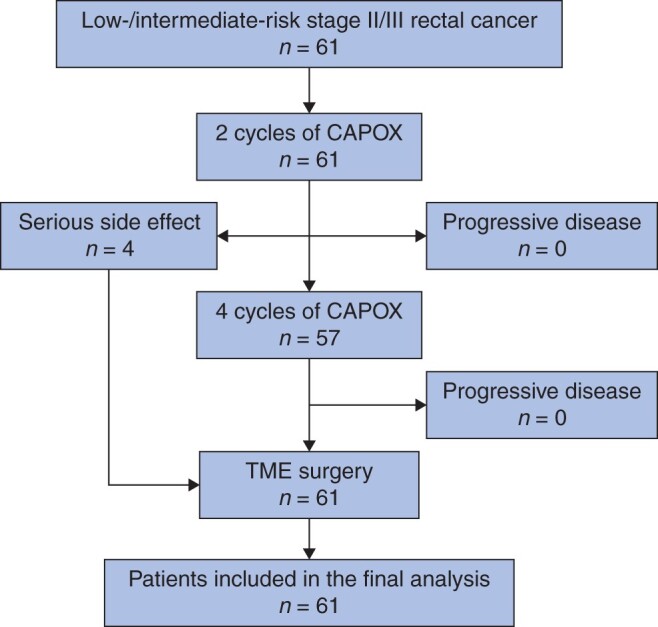
Study flow chart. TME, total mesorectal excision.

From December 2017 to January 2020, a total of 61 eligible patients (47 men and 14 women) were enrolled consecutively (*Fig.  1*). The basic characteristics of the enrolled patients are presented in *Table 1*. The median age of the included patients was 61 (range 29–74) years. Seven patients (12 per cent) had positive extramural venous invasion. The median distance of the tumour from the anal verge was 5.5 (range 1.1–9.8) cm.

**Table 1:** Summary of patient characteristics

Characteristic	Patients (*n *=* *61)
Age (years)*	61 (29–74)
**Gender, male**	47 (77)
BMI (kg/m²)*	23.5 (17.4–34.9)
**ECOG**	
0	35 (57)
1	26 (43)
**Co-morbidity**	
Hypertension	12 (20)
Diabetes	4 (7)
Chronic pulmonary disease	10 (16)
History of cancer	1 (2)
Others	3 (5)
CEA (ng/ml)*	3.2 (0.7–29.3)
CA19-9 (U/ml)*	8.8 (0.6–58.2)
Depth of mesorectal invasion (mm)*	2.2 (0.1–7.4)
**Positive EMVI**	7 (12)
Distance of the tumour from the anal verge (cm)*	5.5 (1.1–9.8)
**Lower border of the tumour**	
Above the peritoneal reflection	4 (7)
At the peritoneal reflection	21 (34)
Below the peritoneal reflection	36 (59)
**Clinical stage**	
T3aN0	1 (2)
T3aN1	7 (12)
T3bN0	20 (33)
T3bN1	23 (38)
T3cN1	2 (3)
T4aN0	2 (3)
T4aN1	6 (10)

Values in parentheses are percentages unless indicated otherwise;

*values are median (range). ECOG, Eastern Cooperative Oncology Group; CEA, carcinoembryonic antigen; CA, carbohydrate antigen; EMVI, extramural venous invasion.

### Neoadjuvant chemotherapy and perioperative features

NCT was discontinued in two patients after three cycles and another two patients after two cycles of CAPOX owing to grade 3/4 adverse effects. The remaining 57 patients (93 per cent) received a full course of CAPOX. No patient was converted to NCRT owing to clinical PD after early or late evaluation (*Fig.  1*). Thirteen patients (2 per cent) experienced grade 3/4 chemotherapy-related adverse effects (*Table 2*). All patients underwent R0 resection with a median operative time of 160 (range 110–420) minutes and a postoperative complication rate of 15 per cent. In particular, four patients (7 per cent) had Clavien–Dindo grade III complications^22^, including three with anastomotic leaks and one with haemorrhage. The median duration of postoperative hospital stay was 7 (range 4–17) days, and no patient died within 30 days after surgery.

**Table 2:** Summary of neoadjuvant chemotherapy and perioperative characteristics

Characteristics	Patients (*n *=* *61)
**Cycles of CAPOX**	
4 cycles	57 (93)
3 cycles	2 (3)
2 cycles	2 (3)
**Complication of chemotherapy**	
Grade I	20 (33)
Grade II	26 (43)
Grade III	11 (18)
Grade IV	2 (3)
**Grade III/IV adverse events of chemotherapy**	13 (21)
Thrombocytopenia	12 (20)
Neutropenia	1 (2)
Anorexia	1 (2)
Hand–foot syndrome	1 (2)
**Operation**	
Low anterior resection (double-stapling technique)	44 (72)
Low anterior resection (intersphincteric resection)	10 (16)
Extralevator abdominoperineal excision	7 (12)
**R0 resection**	61 (100)
**Protective ileostomy**	15 (29)
**Lateral lymph node dissection**	3 (5)
Operative time (min)*	160 (110–420)
**Postoperative complications**	9 (15)
Clavien–Dindo grade I	4 (7)
Chylous leak	1 (2)
Wound infection	1 (2)
Urinary retention	1 (2)
Anastomotic inflammation	1 (2)
Clavien–Dindo grade II	4 (7)
Lung infection	3 (5)
Pelvic abscess	1 (2)
Necrosis of prolapsed colon after Bacon procedure	1 (2)
Clavien–Dindo grade III	4 (7)
Anastomotic leak	3 (5)
Haemorrhage in pelvic cavity	1 (2)
Duration of postoperative hospital stay (days)*	7 (4–17)

Values in parentheses are percentages unless indicated otherwise;

*values are median (range). CAPOX, oxaliplatin 130 mg/m^2^, once daily on day 1, every 21 days, and capecitabine 1000 mg/m^2^, twice daily on days 1 to 14, every 21 days.

### Tumour response

**Fig. 2:**
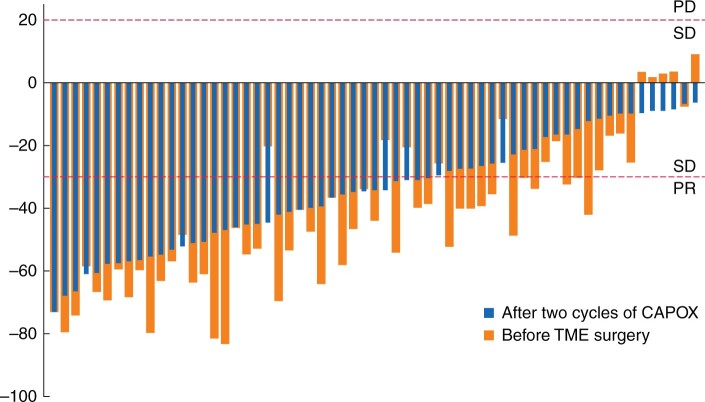
Tumour longitudinal length reduction rate after two and four cycles of CAPOX, ranked by the reduction rate after two cycles CAPOX. CAPOX, oxaliplatin 130 mg/m^2^, once daily on day 1, every 21 days, and capecitabine 1000 mg/m^2^, twice daily on days 1 to 14, every 21 days; PD, progressive disease; SD, stable disease; PR, partial response; the y-axis represents the rate of change in tumour longitudinal length; the upper red dashed line represents the tumour longitudinal length increases by 20%; the lower red dashed line represents the tumour longitudinal length decreases by 30%.

With respect to TLL, 36 (59 per cent) and 44 (72 per cent) patients achieved clinical PR after two and four cycles of CAPOX respectively (*Table 3*). The TLLR rates at early and late evaluation, ranked by the reduction rate, are summarized in *Fig.  2*. A strong correlation (Pearson correlation coefficient = 0.84) was detected for the TLL of each patient after two and four cycles of CAPOX (*P* < 0.001). The TLL of nine patients (15 per cent) increased by more than 10 per cent after four cycles, compared with after two cycles. Only one person had an increase of more than 20 per cent. However, when compared with the baseline, no patient was assessed as having PD after four cycles of CAPOX. A complete mesorectal plane was achieved in all patients. The percentages of pTRG0, pTRG1, pTRG2, and pTRG3 were 21, 8, 43 and 28 per cent respectively. Combining clinical and pathological criteria of tumour regression, 48 patients (79 per cent) were classified as clinicopathological responders.

**Table 3:** Summary of tumour regression and pathological outcomes

Outcomes	Patients (*n *=* *61)
**Clinical partial response**	
After 2 cycles of CAPOX	36 (59)
After 4 cycles of CAPOX	44 (72)
**mrTRG after 2 cycles of CAPOX**	
2	1 (2)
3	26 (43)
4	28 (46)
5	6 (10)
**mrTRG after 4 cycles of CAPOX**	
2	7 (12)
3	28 (46)
4	21 (34)
5	5 (8)
**Complete mesorectum plane**	61 (100)
**Pathological stage**	
T0N0	12 (20)
T0N1	1 (2)
TisN0	1 (2)
T1N0	4 (7)
T2N0	22 (38)
T2N1	2 (3)
T2N2	1 (2)
T3N0	14 (23)
T3N1	4 (7)
**pTRG**	
pTRG0	13 (21)
pTRG1	5 (8)
pTRG2	26 (43)
pTRG3	17 (28)
**Positive circumferential resection margin**	1 (2)
**Perineural invasion**	11 (18)
**Vascular invasion**	4 (7)
Lymph nodes harvested*	15 (3–36)
**Clinicopathological response**	48 (79)

Values in parentheses are percentages unless indicated otherwise;

*values are median (range). CAPOX, oxaliplatin 130 mg/m^2^, once daily on day 1, every 21 days, and capecitabine 1000 mg/m^2^, twice daily on days 1 to 14, every 21 days; mrTRG, tumour regression grade on MRI; pTRG, pathological tumour regression grade.

### Survival outcomes

During a median follow-up of 24 months, one patient died of secondary hilar cholangiocarcinoma. The patient developed a cerebral infarction 1 day before the planned anterior resection, and pCR was achieved after surgery. Cholangiocarcinoma was diagnosed 6 months after the surgery with an elevated carbohydrate antigen 19–9 and a mass on CT scan. None of the patients experienced local recurrence. Four patients developed distant recurrence, including three with liver metastasis and one with lung metastasis. All three patients with liver metastasis underwent liver resection, two of them remained disease-free, and one developed lung metastasis. No deaths specific to rectal cancer were observed during the follow-up period.

### Early prediction of tumour response

**Fig. 3:**
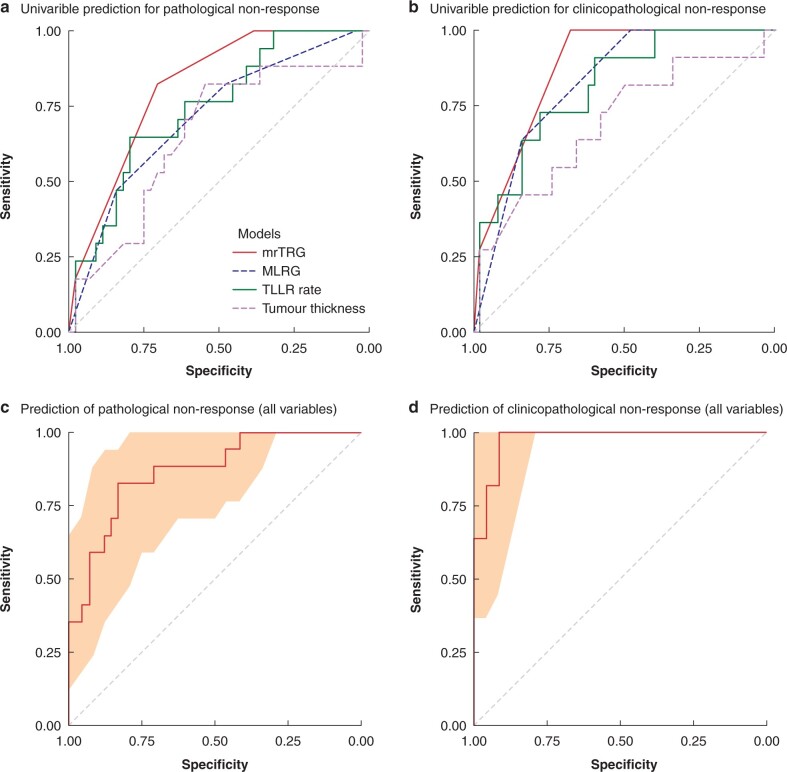
Early prediction of tumour response to neoadjuvant chemotherapy **a** The AUC (area under the curve) of the tumour longitudinal length reduction (TLLR) rate, tumour regression grade on MRI (mrTRG), mucosal lesion regression grade (MLRG) and tumour-thickness reduction rate for predicting pathological tumour regression grade 3 (pTRG3) were 0.74 (95 per cent c.i. 0.60 to 0.88), 0.82 (95 per cent c.i. 0.73 to 0.92), 0.71 (95 per cent c.i. 0.57 to 0.85) and 0.65 (95 per cent c.i. 0.49 to 0.81) respectively. **b** The AUC of the TLLR rate, mrTRG, MLRG and tumour-thickness reduction rate for predicting clinicopathological non-responders were 0.81 (95 per cent c.i. 0.68 to 0.95), 0.88 (95 per cent c.i. 0.82 to 0.95), 0.83 (95 per cent c.i. 0.73 to 0.93, ) and 0.69 (95 per cent c.i. 0.50 to 0.88) respectively. **c** The AUC of the least absolute shrinkage and selection operator (LASSO) regression model for predicting pTRG3 was 0.72 (95 per cent c.i. 0.68 to 0.80). **d** The AUC of the LASSO regression model for predicting clinicopathological non-responders was 0.89 (95 per cent c.i. 0.88 to 0.92). The shadings in **c** and **d** are 95% confidence intervals of the ROC curves.

The use of clinical and radiographic parameters was explored at early evaluation to predict late tumour response after completing NCT. Clinical and radiographic features at early evaluation, including TLLR rate, mrTRG, MLRG and tumour thickness, were analysed to predict pathological tumour response (pTRG0–2). The AUCs of TLLR rate, mrTRG, MLRG and tumour-thickness reduction rate to predict pathological response were 0.74, 0.82, 0.71 and 0.65 respectively (*Fig.  3a*). The above-mentioned variables were also investigated with respect to their predictive performance in clinicopathological responses. AUCs of 0.81, 0.88, 0.83 and 0.69 were observed for the TLLR rate, mrTRG, MLRG and tumour-thickness reduction rate respectively (*Fig.  3b*).

To explore further the combined predictive value of these early evaluation features and MRI textures, a LASSO regression model was fitted to screen for features. One of the first-order features was selected, along with the morphological variables mentioned above, to develop a multivariable logistic model. An AUC of 0.72 (95 per cent c.i. 0.68 to 0.80) was observed for pathological response in the model including TLLR, mrTRG, MLRG and tumour-thickness reduction rate on TRUS (*Fig.  3c*). An additional MRI texture was selected into the multivariable model for clinicopathological response and it yielded an AUC of 0.89 (95 per cent c.i. 0.88 to 0.92) (*Fig.  3d*).

## Discussion

This study demonstrated the effect of NCT alone in patients with low- and intermediate-risk stage II/III rectal cancer and explored predictors for early evaluation of tumour response. An acceptable clinicopathological response rate (79 per cent) and pCR rate (21 per cent) were identified. With respect to early evaluation, the TLLR rate, mrTRG and MLRG were found to be potential early predictors (AUC greater than 0.7) for treatment response.

A pCR rate of 21 per cent was observed in this study with an additional 8 per cent of patients with TRG1, which was higher than most previous reports of 4–13 per cent^11–13^. Although similar pCR rates (16.7–25 per cent) were reported by other trials^21^^,^^23^^,^^24^, they included targeted therapy, which might have confounded the estimated NCT effects. Moreover, targeted therapy may raise new concerns regarding perioperative safety and higher costs. The current study observed promising rates of clinicopathological response (79 per cent) as well as pathological response (pTRG0–2: 72 per cent), underpinning the efficacy of NCT in tumour downstaging. The FOxTROT trial reported a regression rate of 59 per cent and a non-regression rate of 33.9 per cent^25^; in the present study, the TRG3 rate was slightly lower at 28 per cent. Moreover, the FOxTROT trial included patients with colon cancer who would be fit for adjuvant chemotherapy, including those with high-risk factors and right-sided colon cancer. Patients with a higher tumour burden and right-sided colon cancer were considered to be less responsive to chemotherapy. The present study excluded patients with high-risk factors, which might be the main reason for the absence of PD after two and four cycles of CAPOX. Furthermore, this was a phase II trial that enrolled only 61 participants. It is possible that in a larger cohort study PD might be observed in a few patients during NCT.

The GEMCAD 0801 phase II trial included 46 intermediate-risk rectal cancer patients who received preoperative CAPOX with bevacizumab, and no disease progression was observed^21^. Another phase II trial conducted by Hasegawa and colleagues^23^ found that only one patient (1.7 per cent) had progression. In the present study, only patients with low- and intermediate-risk rectal cancers were enrolled, and no PD was observed; hence, no patients were converted to receive chemoradiotherapy. These results, taken together, suggest that the rate of disease progression during NCT might be very low in patients with rectal cancer without high-risk factors. When comparing TLL after two cycles of CAPOX, an increase in TLL over 10 per cent was observed in nine patients after four cycles, and in one patient the increase was more than 20 per cent. However, all nine patients were diagnosed with clinical stable disease after four cycles of CAPOX. Thus, these patients did not receive preoperative CRT chemoradiotherapy.

Although a favourable downstaging effect of NCT was observed in the current trial, whether this could translate into better long-term survival, as NCRT does, needs to be explored further. The downstaging effect was well established as a main contributor to the local control benefit of NCRT. Additionally, accumulating evidence has shown that total neoadjuvant treatment almost doubled the pCR rate and subsequently improved disease-free survival compared with standard NCRT^26^, highlighting the systemic control benefit of chemotherapy. Hence, based on the high response rate observed in the present study, there is reason to presume a good local control effect by NCT, along with the potential for better control of metastasis given the earlier systemic intervention and higher completion rate of chemotherapy^27^^,^^28^.

An important area for more extensive use of NCT alone in rectal cancer is to identify non-responders at an early phase since they are very likely to have poorer outcomes as reported by previous literature on NCRT^29^. In the present study, approximately a quarter of the enrolled patients were non-responders. However, to date, existing studies, including two ongoing large-scale trials (registration numbers NCT01515787 and NCT02288195), evaluated tumour response after 12 weeks of NCT (four cycles of CAPOX or six cycles of FOLFOX). One study conducted an early assessment after one cycle of CAPOX using PET-CT; however, it only enrolled 15 patients and PET-CT was too costly to be incorporated into routine use^15^. Earlier prediction of the tumour response, if possible, would enable physicians to change treatment plans for non-responders. At present, the prognosis of non-responders to NCT has been investigated rarely. The PAN-EX study found that non-responders to NCT also tended to show a reduced rate of regression after sequential NCRT^30^. Although subsequent treatment for non-responders remains an untouched area, early identification and accumulated data on the prognosis of this subgroup of patients can undoubtedly better inform future research. The present study identified potential early predictors including TLLR, mrTRG and MLRG. Additionally, the multivariable model, including MRI texture features, also showed encouraging predictive accuracy, which merits further validation in larger samples. For present non-responders, whether conversion to surgery or enhanced multidisciplinary treatment brings more benefits needs to be investigated further.

There has been a growing interest in investigating organ preservation in patients with a complete clinical response after NCRT. The present study observed a relatively high pCR rate, which might offer an opportunity to develop an organ-preservation strategy with NCT alone in selected low- and intermediate-risk rectal cancer. Due to the assumptive benefit of systemic disease control, NCT might be safer than standard NCRT. However, thus far, no evidence has shown whether NCT could induce a long clinical complete response as NCRT does. Adding NCRT to responders may be another promising option instead of TME. The PAN-EX study found that responders to induction NCT were more likely to achieve mrTRG 1/2 after sequential NCRT compared with non-responders^30^. Moreover, the preliminary results of the OPRA trial showed that induction chemotherapy led to a lower organ-preservation rate than consolidation chemotherapy^31^, but the findings remain to be investigated in different strata of patients with varied responses to NCT. In summary, organ preservation, as an encouraging option for patients with a complete clinical response to NCT, remains to be investigated in larger prospective studies.

Some limitations of this study must be highlighted. First, this was a single-arm phase II trial with a small sample size. The effect of NCT needs to be further verified with a larger sample size in future phase III trials. Second, although the trial identified several potential early predictors, these findings were observed based on limited statistical power and should be validated further. Finally, this study had a relatively short follow-up time; therefore, long-term prognostic outcomes of NCT should be noted in the future.

## Funding

This work was supported by Ministry of Science and Technology of the People’s Republic of China (No. 2017YFC0908204), Department of Science and Technology of Sichuan Province (No. 2021YFS0025), 1.3.5 project for disciplines of excellence, West China Hospital, Sichuan University (No. 20HXJS003 and ZYJC21017), 1·3·5 project for disciplines of excellence – Clinical Research Incubation Project, and West China Hospital, Sichuan University (No. 2019HXFH031).

## Supplementary Material

znab388_Supplementary_Data
