# Supplementary material for: Early response to upfront neoadjuvant chemotherapy (CAPOX) alone in low- and intermediate-risk rectal cancer: a single-arm phase II trial
Source: Br J Surg. 2021 Nov 18;109(1):121–8. doi: 10.1093/bjs/znab388 (PMC10364694; doi:10.1093/bjs/znab388)
Supplement: znab388_Supplementary_Data [file znab388_supplementary_data.zip › Supplementary_Table_1.docx]

Supplementary Table 1. Inclusion and exclusion criteria.

| Inclusion criteria |  | Exclusion criteria |
| --- | --- | --- |
| (1) Age from 18 to 75 years old;  (2) Histopathologically confirmed rectal adenocarcinoma located ≤12 cm above the anal verge;  (3) Low/intermediate-risk stage II/III mid-low rectal cancer;  (4) ECOG score < 2;  (5) No history of colorectal surgery, pelvic radiotherapy, and systemic chemotherapy before enrollment;  (6) No history of allergy to fluorouracil and oxaliplatin. |  | (1) Patients suspected of lynch syndrome;  (2) Patients with distant metastasis during the treatment;  (3) With malignances of other organs within the past 5 years;  (4) Pregnant or nursing women;  (5) History of angina pectoris, congestive heart failure, or myocardial infarction within the past 12 months;  (6) Patients with mental disorders;  (7) Patients with severe infection;  (8) History of serious nephropathy;  (9) Patients with digestive tract diseases which affect the absorption of oral chemotherapy drugs;  (10) Subjects participated in other clinical trials within 4 weeks before enrollment. |
