# Supplementary figures and images for: Early response to upfront neoadjuvant chemotherapy (CAPOX) alone in low- and intermediate-risk rectal cancer: a single-arm phase II trial
Source: Br J Surg. 2021 Nov 18;109(1):121–8. doi: 10.1093/bjs/znab388 (PMC10364694; doi:10.1093/bjs/znab388)

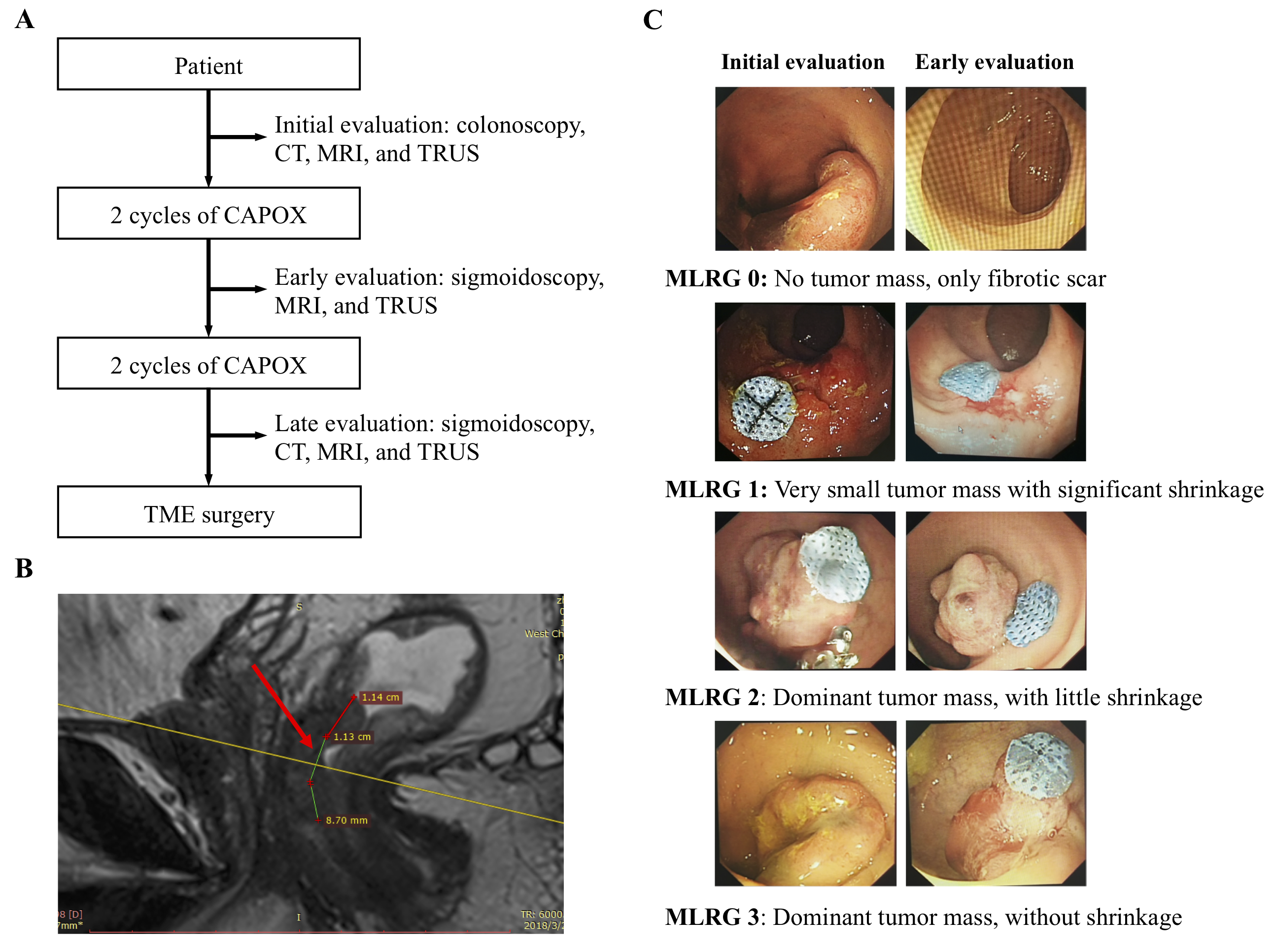

Supplement: znab388_Supplementary_Data [file znab388_supplementary_data.zip › Supplementary_Figure_1.tiff]
